# A highly susceptible hACE2-transgenic mouse model for SARS-CoV-2 research

**DOI:** 10.3389/fmicb.2024.1348405

**Published:** 2024-02-07

**Authors:** Gang Liu, Min Zhang, Baolei Wu, Cheng Zhang, Yan Wang, Xuelian Han, Rongjuan Wang, Li Li, Yuwei Wei, Yali Sun, Xiangwen Cao, Yuan Wang, Yalan Li, Min Li, Guangyu Zhao, Yuehua Ke, Zhendong Guo, Qi Yin, Yansong Sun

**Affiliations:** ^1^State Key Laboratory of Pathogen and Biosecurity, Institute of Microbiology and Epidemiology, Academy of Military Medical Sciences, Beijing, China; ^2^Vanke School of Public Health, Tsinghua University, Beijing, China; ^3^Changchun Veterinary Research Institute, Chinese Academy of Agriculture Sciences, Changchun, China; ^4^College of Veterinary Medicine, Hebei Agricultural University, Baoding, China; ^5^SPF (Beijing) Biotechnology Co., Ltd., Baoding, China; ^6^Beijing Kohnoor Science & Technology Co. Ltd., Beijing, China; ^7^Public Health School, Mudanjiang Medical University, Mudanjiang, China; ^8^Department of Bacteriology, Capital Institute of Pediatrics, Beijing, China

**Keywords:** ACE2, inflammatory response, lung injury, mouse model, SARS-CoV-2

## Abstract

Several animal models have been used to assist the development of vaccines and therapeutics since the COVID-19 outbreak. Due to the lack of binding affinity of mouse angiotensin-converting enzyme II (ACE2) to the S protein of severe acute respiratory syndrome coronavirus-2 (SARS-CoV-2), increasing the susceptibility of mice to SARS-CoV-2 infection was considered in several ways. Here, we generated a COVID-19 mouse model expressing human ACE2 (hACE2) under the control of the CAG promoter. Overexpression of hACE2 did not pose a significant effect on weight growth. After SARS-CoV-2 inoculation, mice showed obvious viral replication and production of inflammation within 7 days, with a gradual decrease in body weight until death. Virological testing found that the virus can replicate in the respiratory system, small intestine, and brain. Additionally, this mouse model was applied to compare two antibody drug candidates, the anti-RBD antibody (MW06) and the mouse CD24-conjugated anti-RBD antibody (mCD24-MW06). Differences in antiviral effects between these two antibodies can be demonstrated in this mouse model when a challenge dose that invalidates the anti-RBD antibody treatment was used. This study provided a new mouse model for studying SARS-CoV-2 pathogenesis and evaluating potential interventions.

## Introduction

COVID-19 involves complex host-pathogen interactions; therefore, it is necessary to develop animal models that can provide measurable readouts for potential interventions (Johansen et al., 2020; Muñoz-Fontela et al., 2020; Villano, 2021; Choudhary et al., 2022). To rapidly evaluate these potential medical countermeasures, such as therapeutic drugs and preventive vaccines, animal models that were susceptible to SARS-CoV-2 infection are invaluable (Zeiss et al., 2021; Choudhary et al., 2022; Fan et al., 2022; Golden et al., 2022; Qi and Qin, 2022; Rizvi et al., 2022; Zhao et al., 2022). However, wild-type mice do not support SARS-CoV-2 replication due to the incompatibility of mouse *ACE2* with the spike protein of SARS-CoV-2 (Yang et al., 2007; Golden et al., 2020; Oladunni et al., 2020). Therefore, several strategies have been developed to genetically modify mice to express the human ACE2 (hACE2) protein on their cellular surfaces, enabling viral replication (Tseng et al., 2007; Arce and Costoya, 2021; Dong et al., 2022; Winkler et al., 2022). Transgenic mice expressing *hACE2* under the control of various promoters, including K18, HFH4, CAG, and human or mouse *ACE2* promoters, have been used to determine susceptibility to SARS-CoV-2 infection and could reproduce some clinical manifestations and pathologies of COVID-19 (Bao et al., 2020; Golden et al., 2020; Jiang et al., 2020; Sun et al., 2020; Asaka et al., 2021; Seo et al., 2022). However, many of these mouse models cannot completely recapitulate the full spectrum of COVID-19 phenotypes, particularly severe clinical symptoms observed in humans, most notably the limited lethality and lack of extrapulmonary manifestations (Knight et al., 2021; Choudhary et al., 2022; Fan et al., 2022). Therefore, it is necessary to develop new mouse models to observe more of these disease manifestations (Fan et al., 2022; Muñoz-Fontela et al., 2022).

**Figure 1:**
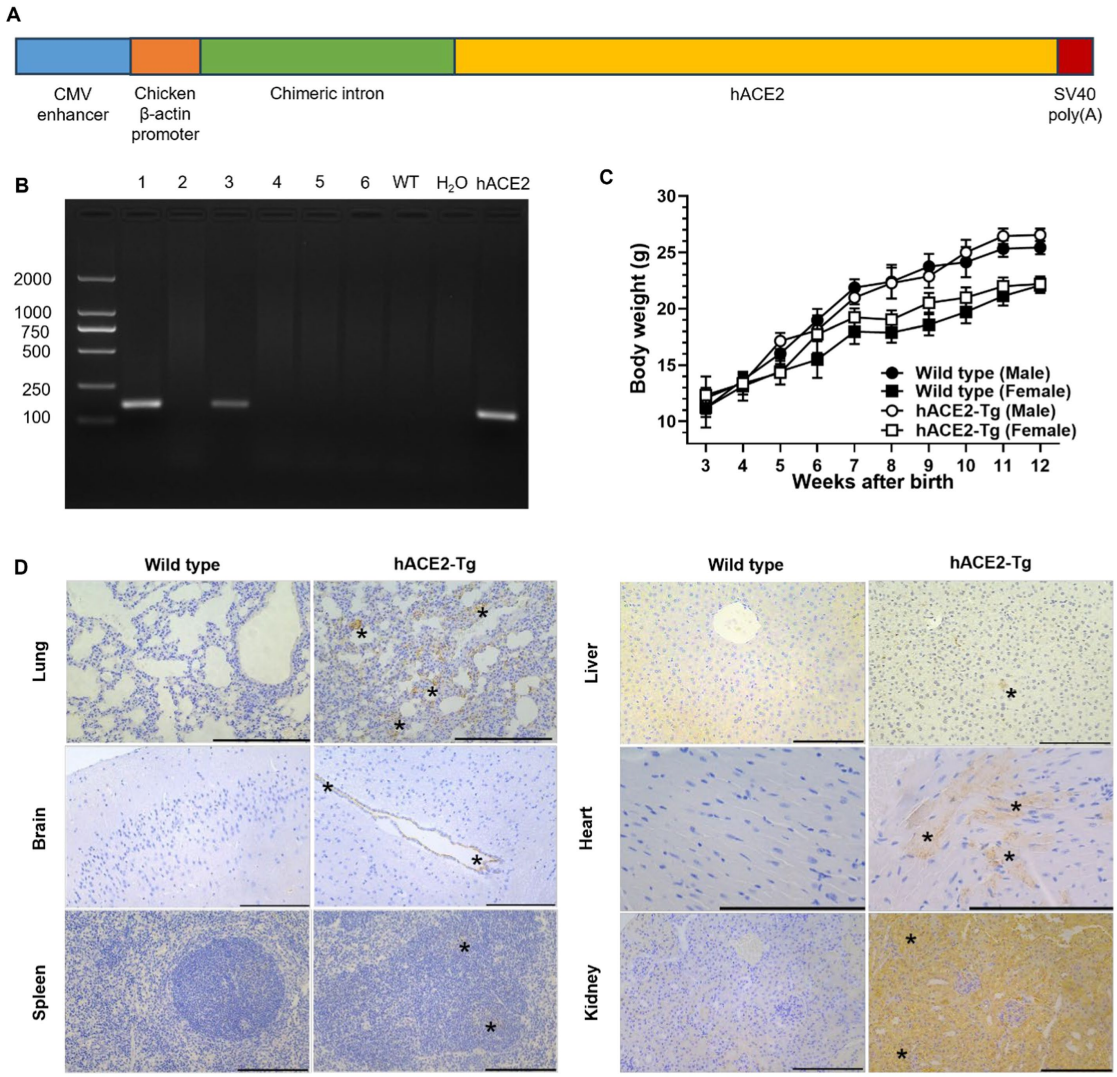
Construction and characterization of a CAG promoter-driven hACE2 transgenic mouse. **(A)** The strategy used to clone gene vectors and identify specific gene constructs using PCR. **(B)** Results of identification with PCR are shown. Lane 1 and 3 indicated two strains of mice were successfully acquired through this strategy. **(C)** Body weight comparison between male and female C57BL/6 and hACE2-Tg mice. **(D)** Immunohistochemical examination of lung, brain, spleen, liver, heart and kidney between C57BL/6 and hACE2-Tg mice. Immunohistochemistry using antibodies against hACE2. Scale bar: 100 μm. In the lung, hACE2 was expressed in alveolar wall capillary endothelial cells (asterisk). In the brain, hACE2 was expressed in ependymal epithelial cells (asterisk). In the spleen, hACE2 was expressed in some cells in the germinal center of splenic corpuscle lymphocytes (asterisk). In the liver, hACE2 was expressed occasionally in individual liver cells (asterisk). In the heart, hACE2 was expressed in myocardial fibroblasts (asterisk). In the kidney, hACE2 was widely expressed in renal tubular epithelial cells (asterisk), and the endothelial cells of glomerular capillaries also showed weak expression of hACE2.

Here, we established a CAG promoter-driven hACE2-expressing mouse model (Figure 1A), which did not show altered weight growth. When exposing our *hACE2* transgenic (hACE2-Tg) mice to SARS-CoV-2, high susceptibility and severe disease phenotype were observed, including high lethality, pathological lesions, and inflammatory responses. The anti-RBD neutralizing antibody and the mouse CD24 (mCD24)-conjugated anti-RBD antibody, respectively named as MW06 and mCD24-MW06, were compared using the hACE2-Tg mouse model as the model. The mouse model can make the effect of the conjugated CD24 molecule be reflected, and when the anti-RBD neutralizing antibody was invalidated by challenge, the mCD24-conjugated anti-RBD antibody showed better effect against anti-RBD antibody, indicating the potential role of CD24 in the fight against COVID-19.

## Materials and methods

### Transgenic mice generation

All targeting vectors were constructed using gene synthesis and standard molecular cloning approaches (Zeng et al., 2016). The *hACE2* coding sequence was used as the template to amplify *hACE2* using the following primers: hACE2-Clon-F: 5′-GAATTCGCCACCATGTCAAGCTCTTC-3′; hACE2-Clon-R: 5′-GCGGCCGCCTAAAAGGAGGTCT-3′. The pCAG-EGFP-N1 plasmid and the amplified *hACE2* product were digested with EcoRI (R3101S, NEB) and NotI (R3189S, NEB) restriction enzymes. The target DNA fragments were subject to gel electrophoresis and recovered by subsequent gel extraction (DP214, Tiangen). The DNA fragments of pCAG-EGFP-N1 and *hACE2* were enzymatically ligated to obtain the pCAG-hACE2-N1 plasmid vector. The pCAG-hACE2-N1 plasmid vector was digested with SpeI (R3133S, NEB) and AflII (R0520S, NEB) restriction enzymes and the linearized DNA fragments were obtained. C57BL/6 mice were purchased from Institute for Laboratory Animal N/A Resources, NIFDC, China. The wide type C57BL/6 mice were used to generated hACE2-Tg mice. The specific targeting sgRNA and Cas9 mRNA were microinjected into wide-type C57BL/6 zygotes, and then these zygotes were subsequently implanted into pseudopregnant mice obtain the founder mice. hACE2-positive founder mice were generated and backcrossed with the wide type C57BL/6 mice to produce generation F1. Starting from F1, hACE2 positive mice in each lineage were mated, and mixing across lineages was not allowed.

### Genotyping

Genomic DNA was extracted from each mouse tail using a tissue DNA extraction kit (DP304, Tiangen) (Zeng et al., 2016). Genotyping was carried out using the following primers: hACE2-F: 5′-TGCTGGTTATTGTGCTGTCTCATCA-3′, and hACE2-R: 5′-CAGGTCTTCGGCTTCGTGGTTAA-3′. Polymerase chain reactions (PCR) were performed using Green Taq Mix (P131, Vazyme). PCR cycling conditions consisted of an initial activation step at 95°C for 15 min, followed by 30 cycles of denaturation at 95°C for 30 s, primer annealing at 58°C for 30 s, and extension at 72°C for 1 min, with a final extension step at 72°C for 7 min. PCR products were visualized on a 1% (w/v) agarose gel and the genotype of each animal was determined.

### Animal infection

All mice involved live SARS-CoV-2 were housed in an approved animal biosafety level-3 facility and given access to standard pellet feed and water *ad libitum*. Mice were anaesthetized intraperitoneally with sodium pentobarbital (50 mg/kg) and infected intranasally with 3 × 10^4^ TCID_50_ (for naive infection) of SARS-CoV-2 (Wuhan strain) in 50 μL of Dulbecco’s Modified Eagle Medium (DMEM) per mouse. Twenty-four 6–8 week-old male hACE2-Tg mice were used in the infection experiment. Mice are randomly assigned to be sacrificed on days 1, 3, and 5 post-infection and for daily monitoring lasting 7–8 days, based on animal age and body weight. Sample sizes were determined on the basis of previous studies and experimental experience. When mice are on the verge of death or unable to move, or do not respond to gentle stimulation, or lose 20% of their pre-experimental body weight, they are considered to have reached the humane endpoint and euthanasia is performed immediately.

### Virus titer detection

Mouse samples for virus titer detection were obtained by the following methods: Mice were dissected at 1, 3, and 5 dpi to collect lung, turbinalia, trachea, liver, brain, spleen, small intestine, kidney, and heart tissues to screen virus titer. Tissues homogenates (1 g/mL) were prepared by homogenizing tissues using an electric homogenizer for 2 min in 2% FBS-DMEM. The homogenates were centrifuged at 3,000 rpm for 10 min at 4°C. The supernatant was collected and stored at −80°C for viral titer detection. Samples were tested for live virus by TCID_50_ assay in a 96-well plate format. Serial 10-fold dilutions of homogenate supernatants were added to 96-well plates of Vero E6 cells. Plates were incubated at 37°C and scored for cytopathic effect at 72 h post-infection. TCID_50_ was calculated using the method of Reed and Muench (Ramakrishnan, 2016).

### Cytokine expression detection

Serum samples for cytokine expression detection were acquired by the following methods: Whole blood was collected by orbital venous puncture and allowed to clot for 30 min at 25°C. Then, samples were centrifuged for 15 min at a force of 2,000 g, and the supernatant was transferred to a new tube. High-throughput liquid-phase protein chip technology was used to detect the cytokine expression in the sera of mice in the uninfected and SARS-CoV-2 infected groups. The experiment was divided into four groups. The first group consisted of sera from three uninfected hACE2-Tg mice, the second group consisted of the sera from 3 to 5 hACE2-Tg mice at 1 day post-infection, the third group consisted of sera from three hACE2-Tg mice at 3 days post-infection, and the fourth group consisted of sera from three or four hACE2-Tg mice at 5 days post-infection. Cytokine detection was performed using an EPX360-26092-901 kit and Luminex 200, according to the manufacturer’s instructions.

### H&E and immunohistochemical analyses

All tissues were immersed in 4% paraformaldehyde for 4–6 h and transferred to 70% ethanol. Individual lobes of tissues were placed in processing cassettes, dehydrated in a serially diluted alcohol gradient, and embedded in paraffin wax blocks (Gu et al., 2020). Before immunostaining, 5-μm-thick tissue sections were dewaxed in xylene, rehydrated in decreasing concentrations of ethanol, and washed in PBS. Then, the tissue sections were stained with H&E. Sections were dehydrated in increasing concentrations of ethanol and xylene.

The 5-μm-thick tissue sections were subject to immunohistochemical analysis by incubating with recombinant anti-ACE2 antibody (ab108209, Abcam) at 4°C overnight. The sections were washed, incubated with horseradish peroxidase-labeled goat anti-rabbit IgG (ZB-2301, ZSBIO, China) for 20 min at room temperature, and then treated with DAB reagent (ZLI-9018, ZSBIO, China). After staining, sections were dehydrated in increasing concentrations of ethanol and xylene (Niu et al., 2018).

### Anti-RBD MAb and mouse CD24-conjugated anti-RBD MAb (mCD24-anti-RBD Mab) preparation

Anti-RBD antibody MW06 was prepared just as before (Jiang et al., 2021). mCD24-anti-RBD antibody, mCD24-MW06, was constructed as follows: the encoding genes of mouse CD24 (1-56aa) were cloned before the N terminal of MW06 light chain genes into the expression vector. The vector of MW06-H and mCD24-MW06-L were transferred into HEK293 cells by using 293fectin (cat: 12347019, Life Technologies) for expression of recombinant mCD24-MW06. Purified MAb proteins were obtained by protein A purification.

### Affinity measurement

Affinity measurement was performed on Octet Red 384 system (Pall ForteBio, United States) using BLI strategy. Antibody (4 μg/mL) was loaded onto the AHC biosensors (Cat. 18–5060, FORTEBIO). Following a short baseline in kinetics buffer, the loaded biosensors were exposed to recombinant S1 of SARS-CoV-2 at concentration of 60 nM and background subtraction was used to correct for sensor drifting. ForteBio’s data analysis software was used to fit the data to a 1:1 binding model to extract the association and dissociation rates. The K_d_ was calculated using the ratio K_off_/K_on_.

### Pseudovirus neutralization assay

The pseudovirus neutralization assays were conducted using Huh-7 cell lines, which were cultured in DMEM with high glucose in a 5% CO_2_ environment at 37°C. The antibodies were diluted in complete DMEM culture media in a 96-well plate, with a total of nine gradients. Subsequently, the virus solution with 1.5 × 10^4^ TCID_50_/mL was added. After 1-h incubation at 37°C and 5% CO_2_, the 96-well plates were seeded with 100 μL of Huh-7 cell (2 × 10^4^ cells/mL). After 48-h incubation, each well was supplemented with 100 μL of Bright-LiteTM luciferase assay system (Vazyme, DD1204-02), and the luminescence was measured using a microplate luminometer (PerkinElmer, Ensight). The IC_50_ were determined by a four-parameter non-linear regression using GraphPad Prism 8.5.

### Antibody evaluation in SARS-CoV-2-challenged hACE2-Tg mice

Mice were intraperitoneally injected with either anti-RBD (MW06) or mCD24-anti-RBD (mCD24-MW06) at a dose of 20 mg/kg body weight (Chi et al., 2022). For prophylactic and therapeutic effect assessment after low-dose viral infections, one day after/before the two antibodies were injected, all mice were intranasally exposed to 2 × 10^3^ TCID_50_ SARS-CoV-2 in 30 μL DMEM per mouse, and the survival and body weight changes in mice were detected. For prophylactic effect evaluation after high-dose viral infections, one day after the two antibodies were injected, all mice were intranasally exposed to 3 × 10^4^ TCID_50_ SARS-CoV-2 in 30 μL DMEM, and the survival and body weight changes were identified. At 5 days after viral exposure, half of the mice were euthanized and equivalent portions of tissues, including lung, trachea, brain, small intestine, liver and heart, were collected for viral titers analysis, and the rest mice were remained for monitoring survival and body weight. Viral exposure was conducted at the ABSL-3 facility.

### Statistics

Three independent biological replicates were used for all experiments unless indicated otherwise. Statistical analysis was performed using GraphPad Prism 8.5. Viral titers were compared among anti-RBD, mCD24-anti-RBD and hACE2-Tg groups of mice and tested for significance of differences by unpaired two-tailed Student’s *t*-tests. One-way ANOVA was performed to compare other groups. Statistical significance was set to *p* < 0.05. p*, *p* < 0.05; p**, *p* < 0.01; p***, *p* < 0.001; p****, *p* < 0.0001.

## Results

### COVID-19 mouse model expressing hACE2 (CAG-hACE2) under control of the CAG promoter

Previous studies have shown that transgenic mice expressing hACE2 are highly susceptible to SARS-CoV-2 infection (Bao et al., 2020; Golden et al., 2020; Jiang et al., 2020). In this study, a COVID-19 hACE2-Tg mouse model overexpressing hACE2 was successfully generated utilizing random integration technology. Genotyping results showed that *hACE2* was successfully inserted in two lines of hACE2-Tg mice (Figure 1B). Both two lines were used for next reproduction, and after several generations, both two showed similar phenotypes with the wide type mouse. In addition, line 1 demonstrated a higher clarity of the target band compared to line 3. This observation suggested a greater abundance of target DNA fragments in line 1, thereby line 1 was chosen to reproduce and establish the hACE2 overexpression lineages. Also, there were no obvious abnormalities in the weight growth of hACE2-Tg mice compared with that of C57BL/6 mice, suggesting transgene has no effect on the growth and development of mice (Figure 1C). Immunohistochemical results showed that the hACE2 protein was highly expressed in some tissues, such as the lungs, heart, and kidneys, while other tissues express lower hACE2 protein, including spleen, brain and liver (Figure 1D). All the above results showed successful introduction of *hACE2* into C57BL/6 mice, which were consistent with other mouse models previously reported (Niu et al., 2018; Gu et al., 2020).

### Susceptibility of CAG-hACE2 mice to SARS-CoV-2 infection

**Figure 2:**
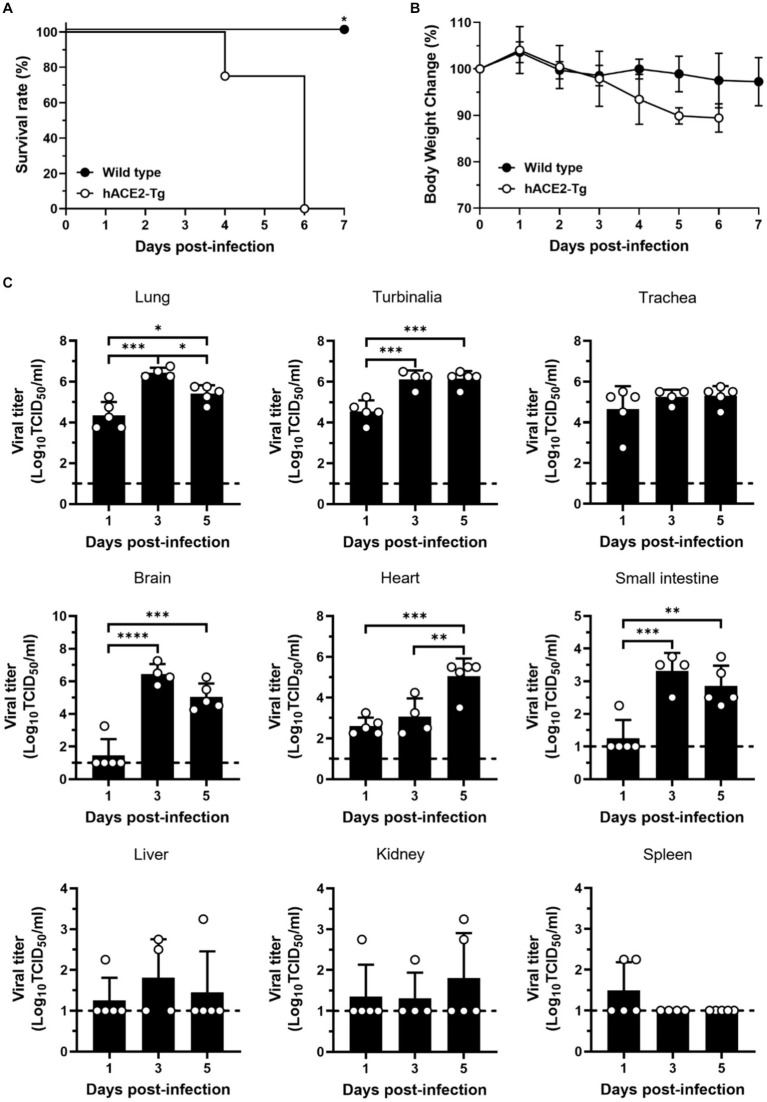
Susceptibility of hACE2-Tg mice to SARS-CoV-2 infection. **(A)** Survival curve of C57BL/6 and hACE2-Tg mice (*n* = 4 per group). **(B)** Changes of body weight of C57BL/6 and hACE2-Tg mice after SARS-CoV-2 infection (*n* = 4 per group). **(C)** Viral titers were detected in tissues of lung, turbinalia, trachea, liver, brain, spleen, small intestine, kidney, and heart. Each dot in the figure represents the outcome for one mouse.

Then, hACE2-Tg mice were infected with SARS-CoV-2 to study the pathogenicity of SARS-CoV-2. First, hACE2-Tg mice were challenged with SARS-CoV-2 at doses of 3 × 10^4^ TCID_50_ virus via intranasal inoculation. Survival and body weight change were recorded and mice were euthanized at various days post-infection (dpi) to measure infectious viral titers as well as to determine pathology change. Compared with C57BL/6 mice, hACE2-Tg mice began to die at 4 dpi and all mice died at 5 dpi, suggesting that hACE2-Tg mice were highly susceptible to SARS-CoV-2 (Figure 2A). We found that hACE2-Tg mice infected with SARS-CoV-2 showed dramatic loss of body weight starting from 4 dpi. About 10–15% body weight was lost for most hACE2-Tg mice (Figure 2B).

To explore viral replication in the hACE2-Tg mice, 9 kinds of tissue samples, including lung, turbinalia, trachea, live, brain, spleen, small intestine, kidney, and heart, were collected from hACE2-Tg mice challenged with SARS-CoV-2 at 1, 3, and 5 dpi. Viral titers were determined by TCID_50_ assay using Vero E6 cells for different tissues from SARS-CoV-2 infected mice. As shown in Figure 2C, viral titers were high in lung, turbinalia, and trachea at just 1 dpi, but not in brain, liver, and small intestine, showing that the respiratory system was the main viral replication site at earlier stages of infection. From 3 dpi, brain and small intestine began to have high viral titers, consistent with respiratory organs including lung, turbinalia, and trachea, indicating that virus gradually transmitted to the nervous system and the digestive system from the respiratory system. It was worth noting that viral titers in spleen were very low, particularly for 3 and 5 dpi, which might result from that large amounts of immune cells in spleen degrade live viral particles.

### Histopathological changes after SARS-CoV-2 infection

**Figure 3:**
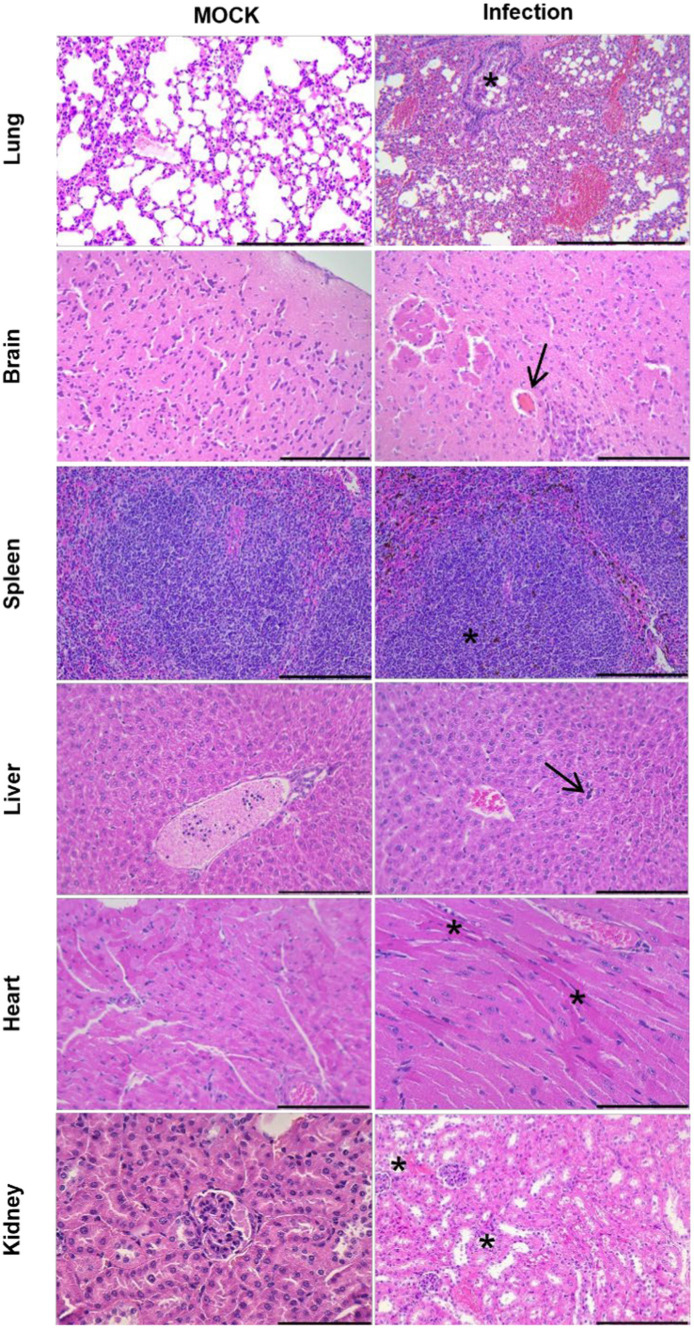
Histopathological examination of uninfected hACE2-Tg mice and hACE2-Tg mice infected with SARS-CoV-2. The lung, brain, spleen, liver, heart, and kidney tissues were evaluated by HE staining. Scale bar: 100 μm. In the lung, the presence of necrosis and shedding of pulmonary bronchial mucosal FIGURE 3 (Continued)epithelial cells, as well as stenosis of the bronchial lumen (asterisk), were observed. Additionally, the alveolar septum exhibited significant thickening, accompanied by a substantial reduction in the number of alveoli. In the brain, transparent thrombus formation within the cerebral microvasculature (arrow) appeared, while no degeneration or necrosis of brain neurons were observed. The spleen displayed enlargement of splenic corpuscles and notable lymphocyte proliferation (asterisk). In the liver, the presence of extramedullary hematopoietic cells (arrow) within the liver lobes was observed, while no hepatocyte degeneration or necrosis was detected. In the heart, mild atrophy of myocardial fibers was identified with concentrated and red stained myoplasma (asterisk). In the kidney, slight dilation of the renal tubular lumen was observed, along with necrotic and detached renal tubular epithelial cells (asterisk).

HE-staining was analyzed from mice infected with SARS-CoV-2 to assess the disease severity (Figure 3). The lungs were the most severely damaged tissue, and its manifestation included diffuse alveolar damage with pneumocyte injury, alveolar hemorrhage, alveolar septal thickening, peripheral parenchymal collapse, and hyaline membrane formation. Extrapulmonary manifestations, namely mild congestion mainly occurred in brain tissue, with other tissues, such as spleen, liver, heart, and kidney tissues, appearing less significant or normal histopathological damage. These findings provided evidence of widespread viral pneumonia and serious acute lung injury in the CAG-hACE2 transgenic mice when infected with SARS-CoV-2 (Winkler et al., 2020; Yinda et al., 2021; Dong et al., 2022; Gawish et al., 2022; Yu et al., 2022).

### Systematic immune response to SARS-CoV-2 infection

**Figure 4:**
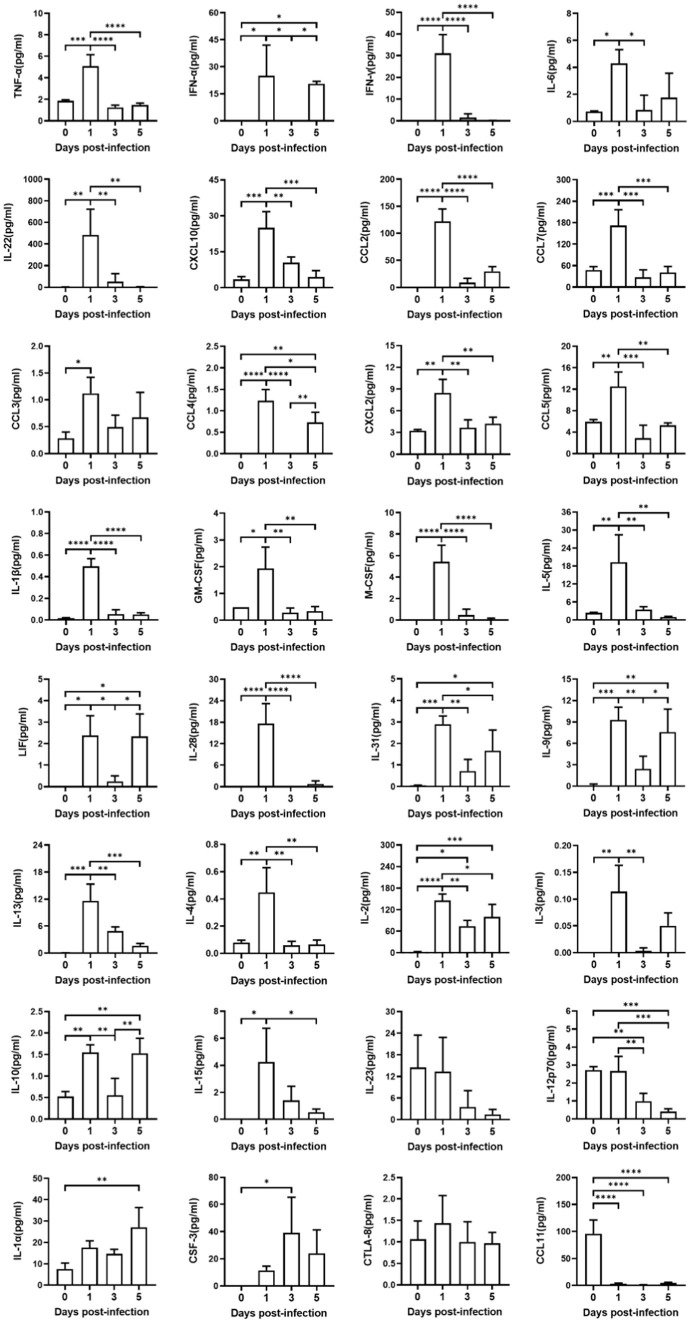
Production of cytokines and chemokines in mice sera infected with SARS-CoV-2. Sera from four groups of mice were detected, including one group of uninfected hACE2-Tg mice, and three groups of infected hACE2-Tg mice with their sera collected at 1, 3, and 5 dpi. Expression of cytokines and chemokines in sera was measured using Luminex 200 assays. One-way ANOVA test was performed to compare groups (*n* = 3–5 per group).

Systematic “cytokine storm” was one of the characteristic pathological features for severely ill patients suffered from COVID-19 (Bishop et al., 2022). To investigate the host immune response of this lethal hACE2-Tg mouse model infected with SARS-CoV-2 infection, we examined expressions of 32 cytokines in the blood samples (Figure 4). At 1 dpi, SARS-CoV-2 infection significantly induced the upregulation of 26 cytokines expression, including tumor necrosis factor-alpha (TNF-α), interferon- alpha (IFN-α), interferon-gamma (IFN-γ), interleukin 6 (IL-6), IL-22, C–X–C motif chemokine ligand 10 (CXCL10), chemokine ligand 2 (CCL2), CCL7, CCL3, CCL4, CXCL2, CCL5, interleukin 1-beta (IL-1β), chemoattractant granulocyte-macrophage colony-stimulating factor (GM-CSF), macrophage colony stimulating factor (M-CSF), IL-5, leukemia inhibitory factor (LIF), IL-28, IL-31, IL-9, IL-13, IL-4, IL-2, IL-3, IL-10, and IL-15. Five cytokines, including IL-23, IL-12p70, IL-1α, CSF-3, cytolytic T lymphocyte-associated antigen 8 (CTLA-8) did not show increasing and one cytokine, CCL11, was downregulated up to zero. These showed that SARS-CoV-2 infection could induce systematic inflammatory responses in hACE2-Tg mouse within a short period, which was consisted with rapid lethal pathogenicity of SARS-CoV-2 in this model. Surprisingly, at 3 dpi and 5 dpi, some key pro-inflammatory cytokines and chemokines, including TNF-α, IFN-γ, IL -22, CXCL10, CCL2, CCL7, CCL3, CXCL2, CCL5, IL-1β, GM-CSF, M-CSF, and IL-5, did not show significant upregulation compared with the uninfected group. These results were consistent with some previous reports (Winkler et al., 2020; Yu et al., 2022), which might indicate the lethality of extensive inflammatory responses immediately after infection.

### The efficacy of antibodies in the prevention and treatment of viral infections

**Figure 5:**
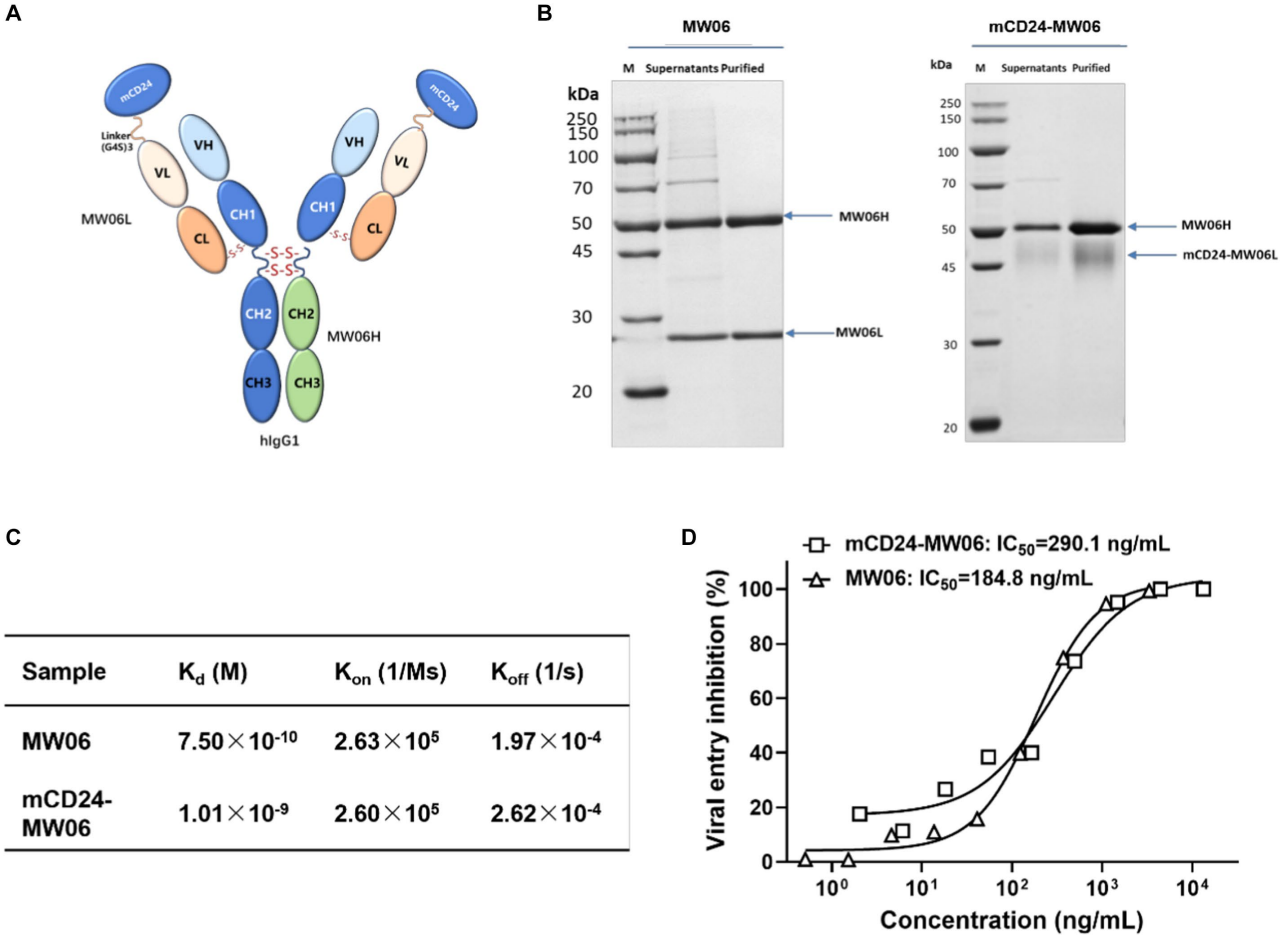
Characterization of the antibody. **(A)** Schematic representation of the structure of mCD24-MW06. **(B)** Protein electrophoresis of two antibodies indicated an increase in the molecular weight of the light chain in mCD24-MW06 compared to MW06, with the size aligning closely with the anticipated theoretical molecular weight. **(C)** Affinity measurement between antibodies and the recombinant S1 of SARS-CoV-2. K_d_, dissociation constant; K_on_, association rate constant; K_off_, dissociation rate constant. **(D)** Neutralization potency of two antibodies against SARS-CoV-2 pseudovirus on Huh-7. The IC_50_ values of MW06 and mCD24-MW06 were determined to be 184.8 ng/mL and 290.1 ng/mL, respectively.

One of the important applications of SARS-CoV-2 infection mouse model was to assess the efficacy of prophylactic and therapeutic interventions, such as chemical drugs and neutralizing antibody treatments (Asaka et al., 2021; Renn et al., 2021). Research findings have demonstrated that CD24 effectively mitigated systemic inflammation and restored immune homeostasis in individuals infected with SARS-CoV-2 (Song et al., 2022). Therefore, we used hACE2-Tg mice to evaluate the protective and therapeutic effects of two antibodies, MW06 and mCD24-MW06. mCD24-MW06 was an antibody constructed on the basis of MW06, and the molecular structure of mCD24-MW06 MAb was schematically shown in Figure 5A. The mCD24 expression gene was located at the N-terminal end of the light chain variable region of MW06. Protein electrophoresis results indicated an increase in the molecular weight of the light chain in mCD24-MW06 compared to MW06 (Figure 5B), with the size aligning closely with the anticipated theoretical molecular weight.

To better characterize antibodies, we have performed experiments to assess their antigen-binding affinities and pseudoviral neutralizing activities. Affinity measurement between antibodies and the recombinant S1 of SARS-CoV-2 was conducted using BLI strategy. As shown in Figure 5C and Supplementary Figure S1, the dissociation constant, Kd, was determined to be 7.50 × 10^−10^ M for MW06 and 1.01 × 10^−9^ M for mCD24-MW06, suggesting that the affinity of both antibodies toward the S1 protein was practically identical. Figure 5D demonstrated the pseudoviral neutralization results of two antibodies. Both antibodies achieved 100% neutralization and neutralized the virus in a dose-dependent manner. The IC_50_ values of MW06 and mCD24-MW06 were determined to be 184.8 ng/mL and 290.1 ng/mL, respectively, indicating that there was minimal disparity in their capacity to neutralize pseudoviruses. The above results indicated that the affinity and neutralizing activity of our developed antibody, mCD24-MW06, were comparable to those of MW06 toward the antigen.

After antibody characterization, the prophylactic and therapeutic effects of the antibodies after low-dose and high-dose viral infections was evaluated. Prophylactic effects of the antibodies after low-dose viral infections (2 × 10^3^ TCID_50_) were first analyzed. As shown in Supplementary Figure S2A, mice that were pre-injected with either MW06 or mCD24-MW06 antibody exhibited survival even at 14 days post-infection. Meanwhile, mice administered with antibodies prior to viral challenge did not demonstrate any significant reduction in body weight (Supplementary Figure S2B). This observation implied that the early administration of antibodies demonstrated effective prophylactic effects after low-dose viral infections. Subsequently, therapeutic effects of the antibodies were assessed and the results were shown in Supplementary Figures S2C,D. Following viral infection, all mice that received the antibody treatment exhibited 100% survival rate after a 14-day period. Moreover, the administration of the antibody effectively ameliorated the weight loss of the mice. The above results suggested that our mCD24-MW06 antibody demonstrated proper prophylactic and therapeutic effects following low-dose viral infections.

**Figure 6:**
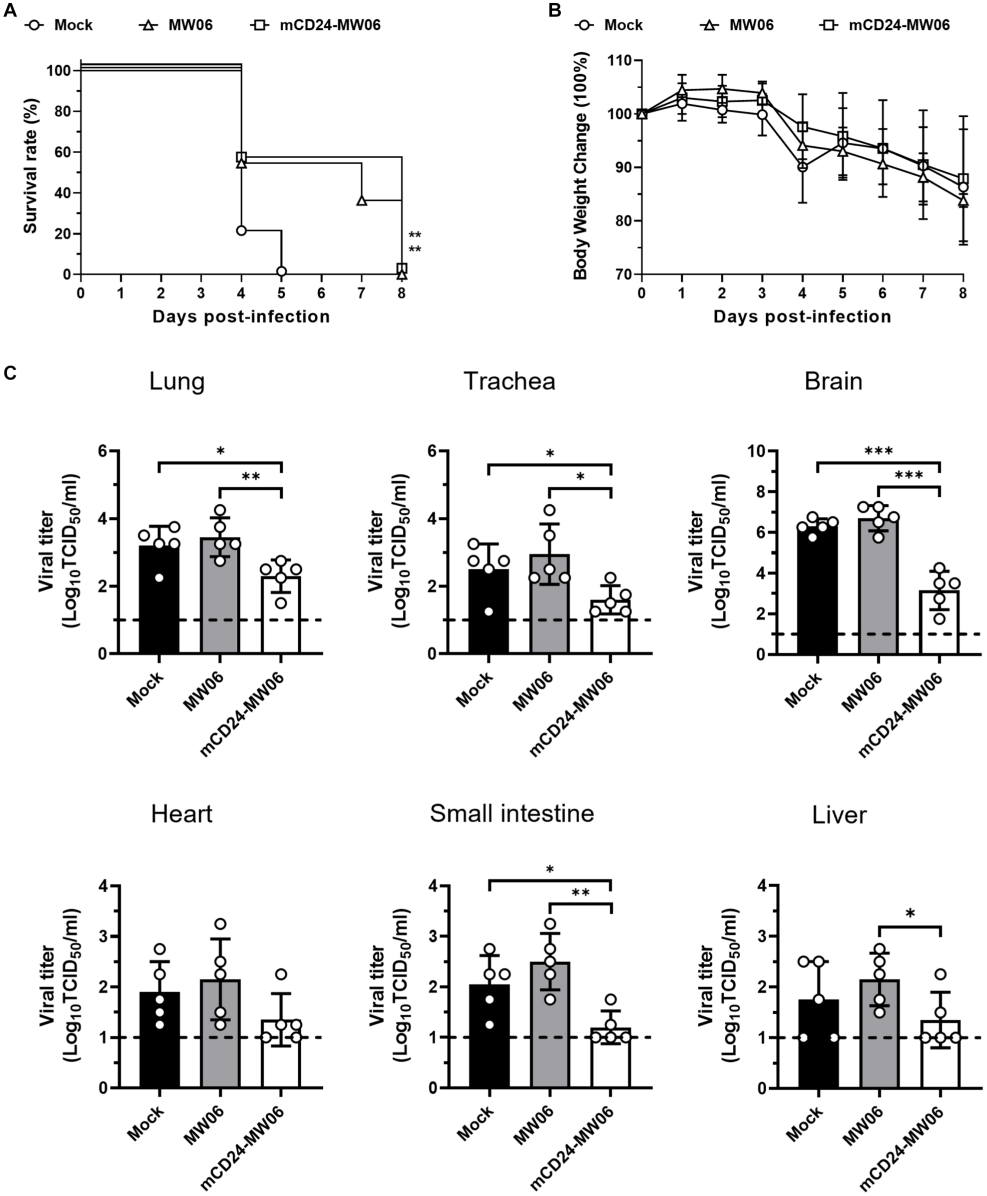
Prophylactic effects of two antibodies, MW06 (RBD antibodies), and mCD24-MW06 (CD24-conjugated RBD antibodies), against high-dose SARS-CoV-2 infection. **(A)** Survival curve of hACE2-Tg mice treated with MW06 or mCD24-MW06. And the survival rate of mice treated with two kinds of antibodies was higher than that of non-treated group, **p < 0.01 (*n* = 10–11 per group). **(B)** Changes of body weight of hACE2-Tg mice treated with MW06 or mCD24-MW06 before SARS-CoV-2 infection (*n* = 11–12 per group). **(C)** Viral titers at 5 dpi in tissues of in lung, trachea, small intestine, heart, liver, and brain tissues. Each dot in the figure represents the outcome for one mouse.

Next, an investigation into the prophylactic effects of antibodies after high-dose viral infections (3 × 10^4^ TCID_50_) was conducted. As shown in Figures 6A,B, when mice were exposed to a high dose of virus (3 × 10^4^ TCID_50_), all mice in mock died on day 5 post-infection, whereas those mice treated with MW06 or mCD24-MW06 injections both died on day 8 post-infection. These findings indicated that antibody injection effectively extended the survival time in the face of a high viral load, yet failed to confer protection against mortality. Although the lethality was not significantly improved for mCD24-MW06 treatment, viral titers were significantly reduced in tissues including lung, trachea, brain, small intestine, and liver, while those in heart were similar between MW06 and mCD24-MW06 treatment (Figure 6C). These findings revealed that hACE2-Tg mice could be used as a tool to assess therapeutic potentials of treatments with neutralizing antibodies or plasma from convalescent patients after infection.

## Discussion

In this study, an *hACE2* transgenic mouse model was developed in which hACE2 expression was driven by the CAG promoter. Analysis of the weight growth showed that overexpression of hACE2 in C57BL/6 mice did not affect their normal physiological status. Infection of these mice with SARS-CoV-2 revealed high susceptibility and obvious pulmonary pathobiological manifestations with dramatic systematic inflammatory responses, in addition to high levels of viral titers. Furthermore, two antibodies were used to validate whether hACE2-Tg mice could serve as an animal model for prophylactic studies with antibodies. MW06 or mCD24-MW06 were able to protect the mice from SARS-CoV-2 to some extent.

Recently, a study reported the development of a CAG promoter-driven hACE2 expressing mouse model (Asaka et al., 2021). This CAG-hACE2 transgenic mouse and the one in the current study showed a similar lethal viral dose. This infection dose was similar to, or potentially even lower than, that of other transgenic mouse models, such as hACE2-expressing mice controlled by the K18 and HFH4 promoters, which were presently the prevailing promoters employed in SARS-CoV-2 infection studies (Jiang et al., 2020; Moreau et al., 2020; Rathnasinghe et al., 2020; Winkler et al., 2020; Yinda et al., 2021). Higher expression of hACE2 in lungs or other organs provided routes for SARS-CoV-2 to enter these organs, resulting in higher morbidity or mortality levels (Hamming et al., 2004; Li et al., 2020).

Although CAG promoter-driven hACE2 expression is exhibited in multiple organs, the surface expression of the ACE2 protein on lung-bronchial and alveolar epithelial cells was most notable, which could explain the high susceptibility observed in this mouse model (Asaka et al., 2021; Seo et al., 2022). Analysis of weight growth revealed that the CAG-hACE2 mouse was similar to that of the non-genetically-modified mouse, indicating that overexpression of hACE2 did not affect normal growth and development of the transgenic mice. This mouse model may be valuable for evaluating the efficacy of vaccines and therapeutics (Asaka et al., 2021; Seo et al., 2022).

Other animal models, including hamsters, ferrets, and monkeys, exhibit natural susceptibility to SARS-CoV-2 and manifest a temporary respiratory disease. Consequently, the wild-type hamster and ferret models have been used extensively to evaluate SARS-CoV-2 countermeasures and disease features. However, mice remain the most efficient and cost-effective animal models capable of reproducing the most severe aspects of COVID-19. Although our transgenic mouse model may not fully capture the complexity and diversity of the human brain, and the extent of infection observed in this model may not reflect the reality of SARS-CoV-2 infection in humans, our hACE2-Tg mouse serves as an *in vivo* model to assess the effectiveness of antiviral treatments in the brain, as well as investigate the mechanisms underlying viral interactions.

A limitation of this study includes the lack of identifying the exact localization and copy numbers of the *hACE2* insertions in the mouse genome, which would be helpful for clarifying why this mouse model was so susceptible to SARS-CoV-2 infection (Knight et al., 2021). The relationship between progressive pathological manifestation and SARS-CoV-2 dose also needs to be further investigated (Winkler et al., 2020). Although a lethal phenotype with severe pulmonary injury was obvious, it was not determined if these mice died mainly from lung dysfunction and respiratory disease, and to what extent other organ injuries contributed to their death (Choudhary et al., 2022).

While hACE2-expressing mouse model exhibited various aspects of respiratory disease and pathology induced by SARS-CoV-2 in humans, it is crucial to acknowledge the limitations associated with animal model when designing studies. To ensure the maximum translational potential of the research, it is important to identify both the similarities and differences between the animal model and humans. Given that no single animal model encompasses all aspects of COVID-19 infection in humans, the choice of the most suitable species will depend on the specific objectives of the study. Various practical factors should also be taken into account, such as animal size, housing conditions, costs, and the availability of suitable reagents. For instance, while non-human primates (NHP) may be more suitable for assessing the immunogenicity and efficacy of a prospective mucosal vaccine targeting upper respiratory tract infections, hamsters may be better suited for evaluating a potential treatment aimed at mitigating the progression of severe respiratory diseases. Alternatively, the extensive array of immunologic reagents and the convenient accessibility and simplicity of genetic modification render mice a valuable model for conducting comprehensive mechanistic investigations into the pathogenesis and immune response of SARS-CoV-2 infection. The judicious utilization of animal models of SARS-CoV-2-induced disease will facilitate enhanced comprehension of SARS-CoV-2 pathogenesis and the advancement of supplementary preventive measures and therapeutic interventions.

In summary, although the current animal models for SARS-CoV-2 infection are useful, a mouse infection model with high susceptibility, severity, and lethality is still needed for investigating the protective effect of prophylactic and therapeutic countermeasures. Albeit not the firstly reported CAG promoter-driven hACE2-expressing mouse model, this study independently provided a new mouse model with high susceptibility and severity for SARS-CoV-2 infection (Asaka et al., 2021; Seo et al., 2022). This study provides another useful animal model for the fight against COVID-19, which will aid in determining potential preventive and therapeutic medicines.

## Data availability statement

The original contributions presented in the study are included in the article/Supplementary material, further inquiries can be directed to the corresponding authors.

## Ethics statement

The animal study was approved by Animal Experiment Committee of Laboratory Animal Center, Institute of Microbiology and Epidemiology, AMMS (approval number: IACUC-IME-2021-017). The study was conducted in accordance with the local legislation and institutional requirements.

## Author contributions

GL: Conceptualization, Data curation, Investigation, Visualization, Writing – original draft. MZ: Data curation, Formal analysis, Software, Writing – original draft. BW: Conceptualization, Formal analysis, Methodology, Software, Writing – original draft. CZ: Formal analysis, Project administration, Resources, Validation, Writing – original draft. YanW: Investigation, Methodology, Supervision, Writing – original draft. XH: Data curation, Formal analysis, Validation, Writing – original draft. RW: Formal analysis, Project administration, Writing – original draft. LL: Resources, Software, Supervision, Writing – original draft. YuwW: Formal analysis, Methodology, Visualization, Writing – original draft. YalS: Methodology, Validation, Writing – original draft. XC: Data curation, Supervision, Writing – original draft. YuaW: Project administration, Resources, Writing – original draft. YL: Data curation, Methodology, Writing – original draft. ML: Project administration, Supervision, Writing – original draft. GZ: Conceptualization, Resources, Supervision, Writing – review & editing. YK: Conceptualization, Project administration, Resources, Writing – original draft. ZG: Conceptualization, Investigation, Resources, Supervision, Writing – original draft. QY: Conceptualization, Project administration, Visualization, Writing – review & editing. YanS: Conceptualization, Funding acquisition, Project administration, Resources, Supervision, Writing – review & editing.
