# Supplementary material for: A highly susceptible hACE2-transgenic mouse model for SARS-CoV-2 research
Source: Front Microbiol. 2024 Feb 7;15:1348405. doi: 10.3389/fmicb.2024.1348405 (PMC10883650; doi:10.3389/fmicb.2024.1348405)
Supplement: Supplementary file 1 [file Image_1.PDF]

A highly susceptible hACE2-transgenic mouse model for SARS-CoV-2 research

Gang Liu[1†], Min Zhang[1†], Baolei Wu[2†], Cheng Zhang[3,4], Yan Wang[5], Xuelian Han[1], Rongjuan Wang[6], Li Li[5], Yuwei Wei[1], Yali Sun[1,7], Xiangwen Cao[1,7], Yuan Wang[1], Yalan Li[6], Min Li[1], Guangyu Zhao[1,7], Yuehua Ke[8*], Zhendong Guo[3,4*], Qi Yin[1*], Yansong Sun[1*]

[1]State Key Laboratory of Pathogen and Biosecurity, Institute of Microbiology and Epidemiology, Academy of Military Medical Sciences, Beijing, China

[2]Vanke School of Public Health, Tsinghua University, Beijing, China

[3]Changchun Veterinary Research Institute, Chinese Academy of Agriculture Sciences, Changchun, Jilin, China

[4]College of Veterinary Medicine, Hebei Agricultural University, 2596 Lucky South Street, Baoding, Hebei, China

[5]SPF (Beijing) Biotechnology Co., Ltd. Beijing, China

[6]Beijing Kohnoor Science & Technology Co. Ltd, Beijing, China

[7]Public Health School, Mudanjiang Medical University, Mudanjiang, China

[8]Department of Bacteriology, Capital Institute of Pediatrics, Beijing, China

***Correspondence:**
Yansong Sun, sunys@qq.com; Qi Yin, jnyzyinqi@163.com; Zhendong Guo, guozd@foxmail.com; Yuehua Ke, yuehuakebj@163.com

[†]These authors contributed equally to this work and share first authorship.

Supplementary figures.

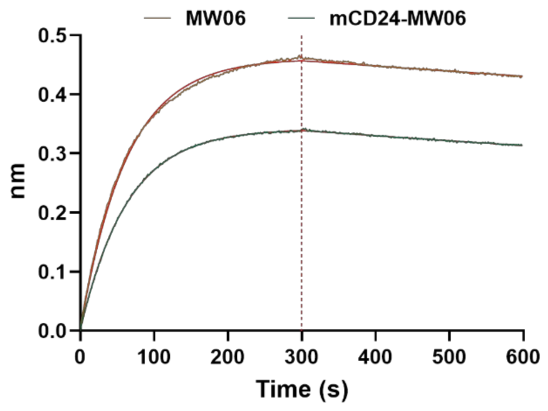

Supplementary Figure 1. The binding kinetics of two antibodies to SARS-CoV S1 recombinant proteins measured by BLI.

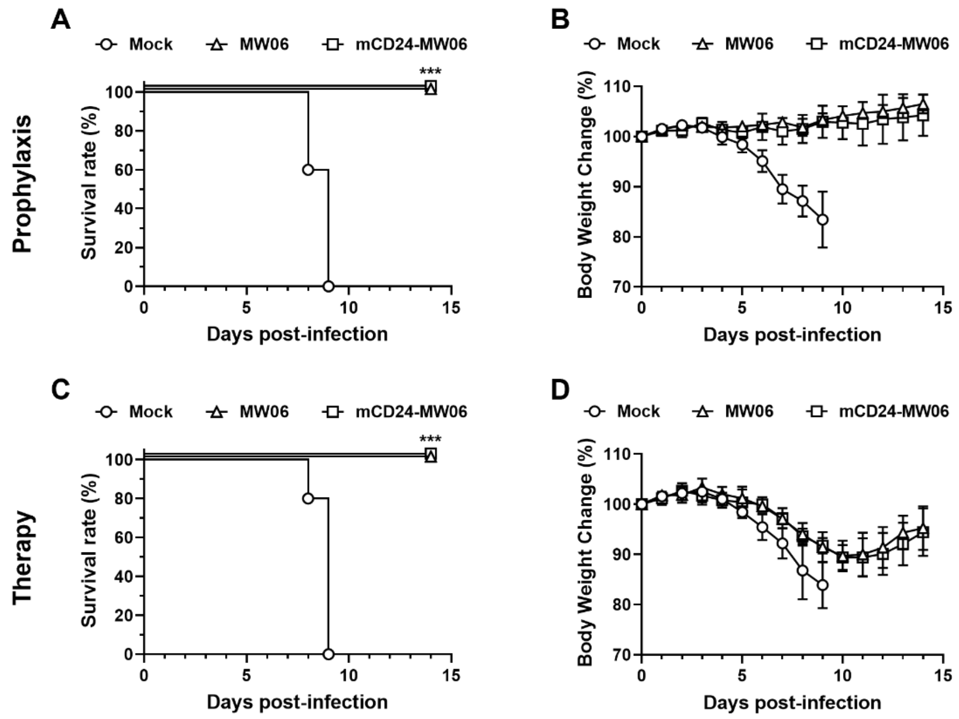

Supplementary Figure 2. Prophylactic and therapeutic effects of two antibodies, MW06 (RBD antibodies), and mCD24-MW06 (CD24-conjugated RBD antibodies), against low-dose SARS-CoV-2 infection. **(A)** Survival curve of hACE2-Tg mice treated with MW06 or mCD24-MW06 before SARS-CoV-2 infection. **(B)** Changes of body weight of hACE2-Tg mice treated with MW06 or mCD24-MW06 before SARS-CoV-2 infection. (n=5 per group). **(C)** Survival curve of hACE2-Tg mice treated with MW06 or mCD24-MW06 after SARS-CoV-2 infection. **(D)** Changes of body weight of hACE2-Tg mice treated with MW06 or mCD24-MW06 after SARS-CoV-2 infection. (n=5 per group).
